# A study of the workforce in emergency medicine in Israel 2012: what has changed in the last decade?

**DOI:** 10.1186/s12245-015-0094-z

**Published:** 2015-12-12

**Authors:** Michael J. Drescher, Zev Wimpfheimer, Aziz Darawsha, Ryan Sullivan, Aviva Goral, Limor Aharonson-Daniel

**Affiliations:** Department of Emergency Medicine, Hartford Hospital, University of Connecticut, Hartford, CT USA; Terem Emergency Medical Centers, Jerusalem, Israel; Emergency Department, Rambam Medical Center, Haifa, Israel; Integrated Residency in Emergency Medicine, University of Connecticut, Hartford, CT USA; Department of Emergency Medicine, Faculty of Health Sciences and PREPARED Center for Emergency Response Research, Ben Gurion University of the Negev, Beer Sheva, Israel

## Abstract

**Objective:**

In 2003, we published a study on the Israeli workforce in emergency medicine (EM). We repeated the study in 2012 to assess changes in the workforce that have occurred in the interval decade.

**Methods:**

This is an observational cross-sectional study of the physician workforce in EM in Israel in 2012. An online survey was sent to the ED medical directors of all general hospitals in Israel querying the numbers of physicians working in the ED, as well as the specialty and level of training of those manning the ED at various times during the day. The workforce in 2012 was compared to that of 2003.

**Results:**

Twenty-four of 28 (86 %) EDs responded. Certified EM specialists have increased from 59 to 164 since 2003. Disparities continue regarding their presence in the ED. Most EM specialists are scheduled during the day whereas they are virtually absent during the night. A total of 58 EM specialists were scheduled countrywide for the weekday day shift and only one overnight. The preponderance of EM specialists working during the day and the large number of supervised and unsupervised residents working at night has not changed substantially since 2003. Eleven departments reported having an EM specialist present during the evenings whereas in 2003, only two departments reported so.

**Conclusion:**

Since 2003, there are more certified EM specialists and more specialist coverage in the ED into the evening hours. Most ED providers are still not emergency physicians, and there is still a preponderance of EM specialist coverage during the day and a lack thereof overnight.

## Background

In 1999, emergency medicine (EM) was officially recognized as a medical specialty in Israel. In 2003, the first certification examinations were held in Israel for physicians who had undergone postgraduate training in EM. That year, we conducted a survey of the physician workforce in EM to understand the status quo and the underlying workforce needs of the country. At that time, we found a relative shortage of certified emergency physicians and that a preponderance of emergency medicine specialists were scheduled during weekday day shifts, leaving the emergency department (ED) staffed in a large part by residents in training for various specialties [1]. Since that time, training programs throughout the country have trained EM physicians in super-specialty programs requiring previous specialization. In 2012, EM was accredited by the Israeli Medical Association Scientific Council as a primary residency and the first such residents enrolled in primary training programs. We elected to do a follow-up survey of the workforce of physicians in emergency departments throughout Israel and to compare the results to those of 2003. Our hypothesis was that in the intervening decade, increased training of, and demand for, emergency physicians would lead to an increase in the level of training and staffing numbers in EDs in Israel. We hypothesized based on anecdotal evidence that the preponderance of EM specialists present during the weekday day shift had not changed.

## Methods

### Setting and population

From June to September of 2012, we performed a study of the workforce of all 28 general hospitals with EDs countrywide in Israel by sending a survey via the Israeli Association for Emergency Medicine, to ED medical directors.

### Survey content and administration

We conducted a survey in the Hebrew language designed to assess the numbers, level of training, and specialty (if any) of physicians working in EDs. We further asked about differential staffing at various times during the day and the week. Additional questions regarding ED and hospital size and ED census were included. The survey was adapted from and designed to replicate a previously published workforce study of the same population in 2003 [1], which had been adapted from a workforce study done previously in the USA [2]. The current survey was adapted for online use and, in contrast to the 2003 survey, was conducted using a secure website. Content differed from the 2003 survey in that several questions regarding nursing staffing were eliminated from the current survey. A question regarding paramedic use in the ED was added. Otherwise, it was similar in content to the 2003 survey. The survey was adapted under the auspices of two of the authors, one of whom has expertise in survey construction. A letter was sent to the directors of EDs in all general hospitals in the country. If surveys were not initially returned, at least two attempts at a telephone call follow-up were done to encourage participation.

### Data analysis

Data were entered using SAS statistical software (SAS Institute Inc., Cary, NC). Simple descriptive statistics are reported. Hospitals were divided into three groups by the number of beds as follows: small (up to 399 beds), medium (400–699 beds), and large (700 beds and up).We stratified the workforce data by physician type, shift, and hospital size. We divided the day into three shifts: morning, evening, and night. The weekend in Israeli hospitals is defined as Friday afternoon through Saturday night.

This study was exempt from Institutional Review Board approval as it did not involve human subjects or patient information.

## Results

Twenty-four of 28 (86 %) survey instruments were returned.

### Characteristics of study subjects

There were seven small, nine medium, and eight large hospitals in the study. Average annual ED census for each group was 54,000, 98,000, and 123,000, respectively.

### Main study results

We found that there were a total of 270 physicians employed full-time by the 24 EDs that responded to the survey. Of the full-time certified 123 EM specialists employed in the ED, 25 were certified in emergency medicine only. Other full-time EM specialists had other specialty certification as well. These included internal medicine, pediatrics, surgery, and orthopedics (see Table 1). In addition to full-time EM specialists, we found 147 physicians from other specialties or with no specialty certification employed full-time in the ED (Table 2) and there were a total of 66 reported part-time physician positions of all types employed in EDs around the country.

**Table 1:** Full-time emergency specialists by additional specialty

Type of specialty	EM only	EM and internal medicine	EM and surgery	EM and pediatrics	EM and orthopedics
Total 123	25	75	7	13	3

**Table 2:** Full-time non-emergency specialists employed in the ED by specialty

Type of specialty	Internal medicine	Surgery	Pediatrics	Orthopedics	Neurology	No specialty (not in training)
Total 147	59	19	6	6	3	54

In comparison, in 2003, we found 140 full-time physicians employed in EDs around the country and another 94 part time.

In characterizing EM specialists, we found that 36 had completed training in the past 5 years. Most EM specialists had done specialty training (as opposed to “grandfathering” in) in Israel whereas only nine completed an EM residency outside of Israel.

As regards training programs, 14 of the departments responding reported having direct postgraduate training programs in EM, with a total of 27 physicians in training (ranging from 0 to 9 residents per program). Seventeen departments reported having a super-specialty training program, with a total of 24 trainees, 18 having had previous training in internal medicine, 4 in pediatrics, and 1 each in surgery and family medicine.

The various departments reported a full-time work week as being from 36 to 45 h with a mean of 39.7 h a week.

**Fig. 1:**
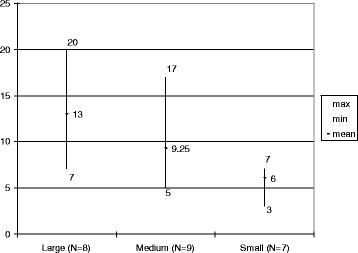
Number of full-time physicians working in the ED grouped by hospital size

**Fig. 2:**
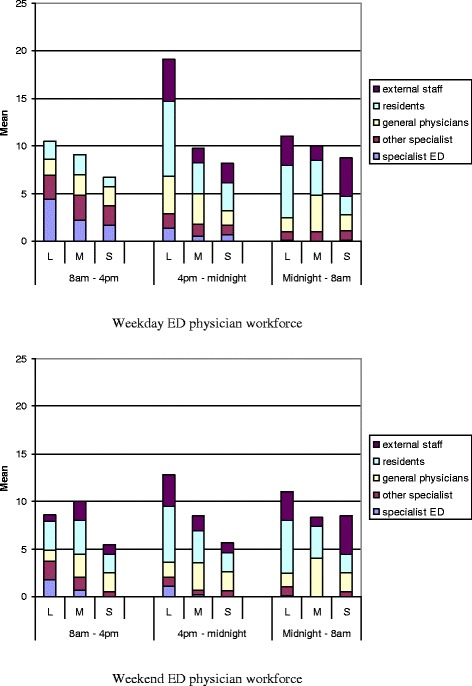
Average physician ED coverage by time of day and level of training in large (*L*), medium (*M*), and smaller (*S*) hospitals, weekdays and weekends

The average number of full-time specialist physicians (of all specialties) employed by the ED—by hospital size—is shown in Fig. 1. These numbers do not include other physicians working in the ED, either “on call” or “covering” the ED for other departments. The number of full-time physicians working in the ED varies by hospital size disproportionately to the difference in ED census, with medium-sized hospitals having the lowest full-time staff-to-visit ratio. The number of physicians actually working (whether belonging to the ED or to other departments) in the ED and the type of physician by level of training and specialty vary from shift to shift and from weekday to weekend (Fig. 2). On an average weekday in Israel, in all responding EDs, there were 58 EM specialists working the day shift, 14 working the evening shift, and 1 EM specialist on the night shift in the country at large.

In comparison in 2003, we found an average of 51 EM specialists scheduled to work the day shift on a typical weekday, 5 EM specialists during the evening shift, and 1.5 EM specialists scheduled for the overnight shift in the entire country on a weekday. Weekends showed a decline in EM specialist coverage compared to weekdays. In the large hospital group, the number of EM specialists scheduled during the day shift during the week was an average of 4.4, whereas at the weekend, it was reduced to an average of 1.8 per department. In the smaller hospital cohort, there were weekends where no EM specialists were reported scheduled for any shift.

#### Limitations

Our study is based on self-reporting from ED medical directors. Although the participants were assured that data would be reported only in the aggregate, and we are not aware of any reason for reporting to be purposefully inaccurate, it is possible that we did not get accurate numbers, for whatever reason, from some EDs. The response rate to our survey was 89 % of emergency departments (24/28). While our data represent the majority of EDs in Israel, the study is lacking data from the remaining 11 %. A previously published study used a similar methodology, albeit with a much lower response rate than ours [3].

As regards pediatric EM, in hospitals where there were both general and pediatric EDs, we did not separately query the pediatric ED medical director. Since many of these departments function with a separate workforce from that of the general ED, we will have not taken many pediatric emergency physicians into account in this survey. This is an area for a future study.

## Discussion

In comparing the current study with that done in 2003, we had a response rate of 24/28 hospitals (86 %) as opposed to 24/25 (96 %) hospitals in the previous survey. However, one participating hospital closed its ED in the interim and the other non-responders were small private hospitals that we attempted unsuccessfully to recruit this time, which were not included in the previous survey. Therefore, the current sample reflects very closely (23/24) the same hospitals that participated in 2003.

Since the last survey performed on the EM workforce in Israel, there was an increase of 93 % in the full-time workforce measured in EDs around the country. In 2012, there were 164 registered specialists in EM in Israel and 71 residents training in EM. This compares to 59 and 37, respectively, in 2003 (personal communication Israeli Medical Association). However, the number of EM specialists reported scheduled for various times of day has not changed substantially, with the large majority of EM specialists scheduled for the weekday day shift. The exception is the evening shift where the presence of senior EM physicians has nearly tripled, from 5 to 14 overall EDs, during a typical weekday. This still works out to an average of less than one EM specialist per ED around the country. Of note, in a report from the Ministry of Health, the percentage of patients arriving to the ED is largest between the hours of 10 AM and 9 PM with peaks at 11 AM and 8 PM [3].

The situation after midnight is even more striking with only one ED reporting scheduling a senior EM physician overnight. Weekend scheduling depends much more on physicians who are in training or who have not completed specialty training. When comparing staffing versus visits, Friday (considered a weekend day in Israel) does have significantly fewer visits. Saturday has a similar amount of visits to a weekday [3]. In smaller hospitals over the weekend, there are no EM specialists reported scheduled. This lack of EM specialists may be especially important if smaller hospitals also have less specialist support in general and on weekends specifically.

Noteworthy also is the fact that during evenings, nights, and weekends, there are external doctors (moonlighters) brought in to staff the ED, whereas during the weekday day shift, none are scheduled, the ED presumably relying entirely on its own staffing.

The shift in the training model in 2012 may impact the understaffing of EM specialists during “off hours”. The direct tract consists of 48 months of training, 30 of which are in the emergency department. Unlike their counterparts who trained previously (or were grandfathered into the specialty), they are unlikely to work in a primary care setting, as their only licensed specialty will be EM. Residents completing this tract will enter the workforce as attending physicians in 2016.

The current survey shows an increase in full-time physicians employed to staff the ED. This should be considered in light of the increase in patient census in EDs throughout Israel since 2003. At that time, it was estimated that there were 2 million visits annually to EDs around the country [1]. Since that time, the population of Israel has grown from 6 to 8 million [4] and the number of ED visits has grown to 2.5 million, excluding obstetrics [5]. This number does not include a sizable number of patients seen at urgent care centers. The largest network of these centers sees 400,000 patients annually with 93 % being discharged home and only 7.8 % being transferred to a hospital-based ED (personal communication). The need for physician coverage is a function not only of new visits but also of throughput time. The total time in the emergency department has increased in Israel between 2009 and 2012 from 2.98 to 3.38 h [3]. Of note, the longest length of stay is in the morning hours which would seem counterintuitive in that attending emergency physicians are present in greater numbers during that period. However, patients often remain in emergency departments from the night shift for discharge by senior physicians in the morning hours.

The importance and relevance of EM specialist presence affect several aspects of emergency care. This is true in regard to their general experience level and regarding specific tasks they perform. When present, senior physicians oversee all medical and trauma resuscitations. Cases which can be discharged home from the ED require review by a senior physician or junior physician at an advanced stage of training. General advice and bedside teaching are part of the senior physicians’ role. On call senior physicians are called to come in to the department in case of excessive patient load, or less commonly a specific complicated patient, or mass casualty incident. Diversion due to crowding, while uncommon, is a decision based on real-time data made together by hospital administration, ED directors, and local ambulance authorities.

It appears clear that there is a shortage of emergency physicians in Israel. Various formulae for calculating the appropriate number of emergency physicians have been proposed and discussed previously in the context of the Israeli EM workforce [1]. More recently, Camargo et al. used a formula for assessing demand for emergency physicians in the USA which assumed that at least one EP should be present 24/7 in each ED. Moreover, a minimum of 5.35 full-time equivalents (FTEs) would be required to staff an ED with single coverage (http://www1.cbs.gov.il/reader/newhodaot/hodaa_template.html?hodaa=201311357). It also assumed that 3548 visits will be seen annually by the average EP, based on 2.8 patients per hour and working 40 h per week, of which 34 % is spent on non-clinical requirements. We can then calculate the demand for EPs for each ED from the formula

$$ \mathsf{Demand} = \mathsf{number}\ \mathsf{of}\ \mathsf{visits}/\mathsf{3548} $$

If we use this equation to obtain a gross estimate of the number of emergency physicians needed to attend to the 2.4 million non-obstetric visits annually in Israel, the number comes to 676 full-time positions. This is a number far larger than what we measured in this survey. Of course, if one changes the assumptions of the equation, in terms of hours worked clinically, or patients seen per hour, the numbers of physicians needed per population would change. For instance, recent changes have reduced the number of clinical hours to less than 40 per week for emergency physicians. There is also the need for essential non-clinical activity including teaching, administration, and research that will further increase the demand for EM physician hours. Given the small number of EM physicians at present in Israel, regardless of the exact formula, and given their distribution by time of day and week, a large number of patients not seen by emergency physicians are being seen by other doctors, from other disciplines at various levels and stages of training.

A shortage of emergency physicians, trained in the discipline, is not unique to Israel. Camargo et al. predicted an ongoing shortage of residency-trained EM physicians in the USA, especially in rural areas, to project into the foreseeable future. Whether or not this well be true in Israel, it is likely that the variance in specialty and level of training of physicians in the EDs shown in our study results in variability in care. Where emergent diagnosis and interventions are most time dependent, examples being acute myocardial infarction, stroke, trauma, and sepsis, best practice should dictate minimizing such variability and increasing the level of training and experience in the ED as much as possible.

## Conclusions

While there has been an increase in the number of FTEs assigned to the ED since 2003, there has also been an increase in the number of visits. There is an increase in the presence of EM specialists during the evening hours compared to 2003. However, it is clear from the preponderance of senior EM physicians present during the weekday day shift, with no EM specialists present overnight, and none in smaller hospitals over the weekend, that EM in Israel continues to work according to a paradigm whereby senior physicians are present during regular working hours, available on call, while “off hours,” physicians in training and to a lesser extent other specialists care for patients in the ED.
